# Ultrasonographic and power doppler parameters of nails fail to differentiate between onychodystrophy in patients with psoriasis vulgaris or psoriatic arthritis

**DOI:** 10.1186/s42358-024-00367-x

**Published:** 2024-04-11

**Authors:** Anber Ancel Tanaka, Betina Werner, Annelise Correa Bueno Bragatto, Thelma Larocca Skare, Bárbara Stadler

**Affiliations:** 1 Dermatology Service, Mackenzie Evangelical University Hospital, Avenida Sete de Setembro 4698 Sala 201, CEP: 80730-320 Curitiba, Paraná Brazil; 2 Post-graduate Program - Internal Medicine and Health Sciences, Federal University of Paraná, Curitiba, Paraná Brazil; 3 Department of Pathology, Clinics Hospital of the Federal University of Paraná, Curitiba, Paraná Brazil; 4 Reumathology Service, Mackenzie Evangelical University Hospital, Curitiba, Paraná Brazil

**Keywords:** Nail, Psoriasis, Ultrasound

## Abstract

**Background:**

Nail involvement is frequent in patients with psoriasis (Pso) and psoriatic arthritis (PsA) and there is a relationship between nail involvement and inflammation of the enthesis. The main objective of the present study is to describe the ultrasound findings and clinical characteristics of nails from patients with psoriasis and psoriatic arthritis with and without nail dystrophy.

**Methods:**

A cross-sectional study including consecutive patients with PsO and PsA was carried out. The study patients were divided into 4 groups, totaling 120 participants. Group 1: patients with psoriasis vulgaris and clinically normal nails; Group 2: patients with psoriasis vulgaris and onychodystrophy; Group 3: patients with psoriatic arthritis and clinically normal nails; Group 4: patients with psoriatic arthritis and onychodystrophy; All patients were submitted to dermatological and rheumatological clinical analysis. Ultrasound examinations was performed by a single examiner, blinded to all clinical data, with ultrasound high resolution, in B-mode or gray-scale (GS), Power Doppler (PD) and Spectral Doppler.

**Results:**

A significant difference was found between the groups regarding the variable Psoriasis Area and Severity Index (PASI) (*p* = 0.008) and body surface area (BSA) (*p* = 0.005), with patients with psoriatic arthritis having lower PASI and BSA compared to patients with only cutaneous psoriasis. A positive relationship was found with the average ultrasound thickness of the nail bed and the Nail Psoriasis Severity Index (NAPSI) in correlation analysis (*rho* = 0.344). When we grouped patients with psoriasis and psoriatic arthritis, there was no significant difference between the cutaneous psoriasis groups and the psoriatic arthritis groups in terms of nail plate GS (*p* = 0.442), nail bed PD (*p* = 0.124).

**Conclusion:**

Greater nail bed thickness indicates early psoriatic nail disease, as confirmed in our study correlating NAPSI with nail bed thickness. Ultrasonography is a low-cost exam, promising in the evaluation, showing that the ultrasound grayscale is consistent with those who have dystrophic nails, but it can’t distinguish psoriasis from psoriatic arthritis, even in those with nail dystrophy.

**Supplementary Information:**

The online version contains supplementary material available at 10.1186/s42358-024-00367-x.

## Introduction

Psoriasis is a common, chronic papulosquamous skin disease that occurs worldwide, presenting at any age and imposing a substantial burden on individuals and society. It is associated with several significant medical conditions, including depression, psoriatic arthritis, and cardiometabolic syndrome [1]. Nail involvement is a common occurrence among patients with psoriasis, present in approximately 56% of cases of psoriasis vulgaris and around 80–90% of individuals with psoriatic arthritis. In 6% of cases, nail involvement may be the only manifestation. Nail psoriasis is correlated with more severe disease, characterized by earlier onset and a higher risk of psoriatic arthritis, and it may be employed as an early diagnostic parameter among patients with psoriasis [2]. 

Psoriatic arthritis, a heterogeneous, inflammatory, musculoskeletal disease that can cause permanent damage to both peripheral and axial joints, is the most common comorbidity of psoriasis [3]. A notable association has been established between nail involvement and inflammation of the enthesis at distal joints, potentially acting as a triggering factor in the development of joint symptoms within the spectrum of psoriatic disease [4]. Because skin symptoms associated with psoriasis often precede psoriatic arthritis, proactive screening of patients with all severities of psoriasis for the signs and symptoms of psoriatic arthritis is key to early diagnosis and intervention [3, 4]. 

Ultrasound (US) has proven its efficacy as a sensitive imaging technique and a valuable tool for assessing inflammatory enthesis involvement in patients with psoriatic arthritis. Ultrasound in nail psoriasis is a diagnostic technique that has been used to assess structural changes in nails associated with psoriasis. Ultrasound is a non-invasive tool that uses high-frequency sound waves to create detailed images of internal body structures. In the context of nail psoriasis, ultrasound can provide valuable information about changes in the nail bed, nail matrix, and surrounding structures [5]. Subclinical inflammations detectable by US has been noted even in psoriasis patients lacking a prior history of arthropathy or psoriatic arthritis, underscoring its potential significance [6]. However, uncertainties persist regarding whether ultrasonographic findings obtained solely through US examination can effectively differentiate between patients with psoriasis devoid of arthritis yet exhibiting nail lesions and those with psoriatic arthritis coupled with nail involvement. The primary objective of this study is to compare the sonographic findings and clinical characteristics of nails in patients affected by psoriasis and psoriatic arthritis, both with and without nail dystrophy. Additionally, we aim to discern potential associations among sonographic findings that might contribute to distinguishing between these distinct populations.

## Methods

A cross-sectional study is being conducted from November 2020 to January 2022, enrolling consecutive patients with psoriasis and psoriatic arthritis based on the CASPAR classification criteria [7]. Participants were recruited from the outpatient Dermatology Service Unit at Hospital Evangélico Mackenzie. Patients who were seen at the outpatient clinic were evaluated consecutively. Only those who met the inclusion criteria and agreed to participate in the research were included.

Inclusion criteria: The selected individuals will consist of adult patients aged 18 and above, with no age cutoff, of both genders. Participants must be volunteers who are well-informed and have agreed to the collection of clinical information and materials after reading and completing the informed consent form. Exclusion criteria were patients who did not provide informed consent, those with positive results in direct mycological exams or nail lesion cultures for fungi and/or bacteria, individuals with a history of trauma in fingernails in the last 6 weeks, local glucocorticoid injections in the distal interphalangeal (DIP) joints, or a diagnosis of osteoarthritis in the hands.

The study adhered to the principles outlined in the Declaration of Helsinki and complied with local regulations. Ethical approval for the study was granted by the Hospital’s Local Ethics Committee and received a favorable opinion for the study to proceed under protocol 4.279.067 on September 15, 2020, and written informed consent was obtained from all participating patients.

Patients diagnosed with psoriasis and psoriatic arthritis were sequentially assessed for age, sex, clinical presentation of psoriasis and psoriatic arthritis, disease duration, presence of nail abnormalities, Psoriasis Area and Severity Index (PASI) scores, Nail psoriasis Severity Index (NAPSI) scores for cases with onychodystrophy, and epidemiological profiles were obtained on the same day, while ultrasound examinations were conducted on a separately scheduled day. The study cohort comprised participants equally divided into four groups, as follows.“: Group 1 - patients with psoriasis vulgaris and no evident nail abnormalities; Group 2 - patients with psoriasis vulgaris and onychodystrophy; Group 3 - patients with psoriatic arthritis and clinically normal nails; Group 4 - patients with psoriatic arthritis and onychodystrophy. All participants from Groups 1 to 4 underwent thorough dermatological and rheumatological clinical assessments.

Ultrasound examinations were conducted on the following nails: Groups 1 and 3 (patients with clinically normal nails): Third finger of both the right and left hands. Groups 2 and 4 (patients with nails with dystrophy): The most dystrophic nails on each hand, both left and right, as determined by NAPSI measurements (thus, two nails per individual).

This approach aimed to comprehensively evaluate the clinical and ultrasound characteristics of patients in various groups, shedding light on the potential differentiating factors between psoriasis and psoriatic arthritis with or without nail involvement.

Ultrasound examinations were conducted by a single examiner, an experienced rheumatologist trained in this imaging technique (B.S). The examiner was blinded to all clinical data during the assessments. Ultrasound imaging was performed using an Mylabel40 model from the Esaote brand (Italy) device, a high-resolution system equipped with B-mode or gray-scale (GS), Power Doppler (PD), and Spectral Doppler capabilities, utilizing a linear transducer with a frequency range of up to 18 MHz. The examinations took place in a climate-controlled room at an approximate temperature of 24 degrees Celsius. The room was semi-lit, and patients were positioned in front of the examiner, separated by a stretcher, with their hands resting on it in a relaxed manner. Patients were instructed not to discuss their medical condition with the operator.

For B-mode nail analysis, the linear transducer operating at an 18 MHz frequency was employed to assess various parameters, including nail bed thickening, undulations, and loss of the trilaminar pattern of the nail layer. To achieve optimal image definition and sharpness, gain adjustments were made. A significant amount of gel was applied to the nail plate to minimize artifacts and enhance image clarity. The examination involved analyzing nail structures in both longitudinal and transverse planes.

Nail bed thickness was measured in the longitudinal plane at the level of the eponychium. A straight line was drawn, referencing the first point on the ventral lamina to the second point on the periosteum. A normal reference value for nail bed thickness was considered to be less than or equal to 2 mm, following established guidelines [8, 9]. 

For the analysis of the nail plate, a semi-quantitative scale was utilized as follows: GS0: Trilaminar pattern preserved; GS1: Slight alteration in the trilaminar pattern, or presence of 1 tortuosity or flaw in the nail. GS2: Intense alteration in the laminar pattern, but some segment of the nail is still preserved; more than one tortuosity or flaw in the nail. GS3: Total loss of the trilaminar pattern, thickening, deformity, or loss of the entire nail shape; severe abnormalities with destruction of the entire nail segment.

Examinations falling within the GS1, GS2, and GS3 criteria of the semi-quantitative scale was deemed as abnormal nail plate.

The analysis of the nail bed was conducted in Power Doppler (PD) mode to quantify blood flow and was achieved using the linear transducer operating at a frequency of 15 MHz, with a Pulse Repetition Frequency (PRF) of 0.7 and a low wall filter. To ensure accurate results, a substantial amount of gel was applied to prevent artifacts and vessel collapse. Like the nail plate analysis, evaluations were performed in both longitudinal and transverse planes.

A semi-quantitative scale for PD was adopted as follows: PD0: Absence of PD signal in any part of the nail bed. PD1: Presence of 1 point or 25% of PD signal in any part of the nail bed, primarily at the nail insertion. PD2: Presence of 2 or 3 isolated points or between 25% and 50% of PD signal in any part of the nail bed, mainly at the nail insertion. PD3: Presence of more than 50% of PD signal across the entire nail bed, particularly at the nail insertion [5, 7, 8]. 

The PD2 and PD3 criteria of the above semi-quantitative scale were considered as indicative of nail bed abnormal blood flow. It is important to note that the nail bed may exhibit physiological vascularization, and therefore PD1 will be considered as normal.

Spectral Doppler measurements were taken at the location with the highest PD signal, and the resistance index was calculated using the equation: minimum peak systolic flow - end diastolic flow divided by systolic flow. This calculation was automatically calculated by the ultrasound device after recording the spectral wave and manually marking the peaks of the systolic and diastolic waves on the displayed curve. A resistance index less than 1 was considered as suggestive of inflammation [8, 9]. 

The data were tested for their distribution using the Shapiro-Wilk test. Descriptive statistics median (minimum-maximum) was employed to summarize the results involving quantitative variables, while categorical variables were expressed in terms of absolute and relative frequencies. For group comparisons, the Kruskal-Walli’s test was employed for quantitative variables and Pearson Chi-square test for categoric variables. Correlation analysis was performed using the Spearman coefficient. The analyzes were carried out using the software Statstica Statsoft 7.0., considering a p-value < 0.05 to indicate statistical significance.

## Results

A total of 120 patients were enrolled in the study, with a balanced distribution of sex and age among individuals with psoriasis and psoriatic arthritis. Within the arthritis groups, 75% exhibited peripheral arthritis, while 25% presented axial arthritis. Significant differences were observed between the groups regarding the PASI variable (*p* = 0.008) and BSA (*p* = 0.005), as indicated in Table 1.

**Table 1:** Distribution between the groups regarding the PASI and BSA

			PASI BSA			
	Group	Mean	n	SD	Minimum	Maximum
PASI	1 + 2	3.0	60	5.79	0	34
	3 + 4	1.5	60	5.44	0	27
BSA	1 + 2	2.0	60	3.70	0	13
	3 + 4	1.0	60	4.22	0	18

PASI: psoriasis area and severity Index. BSA: body surface area n: number participants SD: standard deviation. Group 1 - patients with psoriasis vulgaris and no evident nail abnormalities; Group 2 - patients with psoriasis vulgaris and onychodystrophy; Group 3 - patients with psoriatic arthritis and clinically normal nails; Group 4 - patients with psoriatic arthritis and onychodystrophy. Patients with psoriatic arthritis (groups 3 and 4) demonstrated lower PASI (*p* = 0.008) and BSA values (*p* = 0.005) compared to those with psoriasis alone (groups 1 and 2)

Patients with psoriatic arthritis (groups 3 and 4) demonstrated lower PASI and BSA values compared to those with psoriasis alone (groups 1 and 2). On the other hand, no significant differences were found in terms of NAPSI (*p* = 0.119) between patients with and without arthritis as indicate in Table 2.

**Table 2:** Distribution between the groups regarding NAPSI

		NAPSI			
Group	Average	n	SD	Minimum	Maximum
1		0			
2	28.00	30	17.95	6	97
3		0			
4	22.00	30	12.52	6	52
Total	25.00	60	15.64	6	97

NAPSI: nail psoriasis severity index; n: number; SD: standard deviation

Group 2 - patients with psoriasis vulgaris and onychodystrophy

Group 4 - patients with psoriatic arthritis and onychodystrophy

No significant differences were found in terms of NAPSI (*p* = 0.119)

A positive correlation was identified between the average ultrasound thickness of the nail bed and the Nail Psoriasis Severity Index (NAPSI) through correlation analysis (rho = 0.344). Ultrasound findings revealed that the GS ratio of the nail plate in groups 1 and 3 were similar, while groups 2 and 4, comprising patients with nail dystrophy, displayed clinical similarities. This suggests that there is no discernible distinction in dystrophic nails between individuals with psoriasis alone and those with psoriasis accompanied by arthritis. In terms of Power Doppler results, groups 3 and 4 exhibited resemblances, as did groups 1 and 2. Specifically, when analyzing only patients with nail dystrophy, no significant divergence was noted between cutaneous psoriasis and psoriatic arthritis groups concerning Power Doppler (*p* = 0.16).

Upon grouping patients with psoriasis and psoriatic arthritis, there were no significant differences between the cutaneous psoriasis groups (groups 1 and 2) and the psoriatic arthritis groups (groups 3 and 4) regarding GS nail plate (*p* = 0.442), PD nail bed (*p* = 0.124), and IR E (*p* = 0.758). The gray scale and power doppler results are summarized in the Table 3.

**Table 3:** The gray scale and power doppler results

Degree in percentage	Group 1	Group 2	Group 3	Group 4
Gray Scale (GS)				
0	39 (65.00%)	0 (0.00%)	37 (61.67%)	1 (1.66%)
1	19 (31.67%)	17 (28.33%)	22 (36.66%)	23(38.33%)
2	2 (3.33%)	25 (41.67%)	1 (1.66%)	25 (41.67%)
3	0 (0.00%)	18 (30.00%)	0 (0.00%)	11 (18.33%)
**Power Doppler (PD)**				
0	34 (56.67%)	31 (53.45%)	35 (58.33%)	22 (36.67%)
1	17 (28.33%)	14 (24.14%)	10 (16.67%)	17 (28.33%)
2	9 (15.00%)	12 (20.69%)	15 (25.00%)	19 (31.67%)
3	0 (0.00%)	1 (1.72%)	0 (0.00%)	2 (3.33%)

GS0: Trilaminar pattern preserved; GS1: Slight alteration in the trilaminar pattern, or presence of 1 tortuosity or flaw in the nail; GS2: Intense alteration in the laminar pattern, but some segment of the nail is still preserved; more than one tortuosity or flaw in the nail; GS3: Total loss of the trilaminar pattern, thickening, deformity, or loss of the entire nail shape; severe abnormalities with destruction often entire nail segment.PD0: Absence of PD signal in any part of the nail bed; PD1: Presence of 1 point or 25% of PD signal in any part of the nail bed, primarily at the nail insertion; PD2: Presence of 2 or 3 isolated points or between 25% and 50% of PD signal in any part of the nail bed, mainly at the nail insertion; PD3: Presence of more than 50% of PD signal across the entire nail bed, particularly at the nail insertion. When grouping patients with psoriasis and psoriatic arthritis, no significant differences were observed between the cutaneous psoriasis groups (groups 1 and 2) and the psoriatic arthritis groups (groups 3 and 4) in terms of GS nail plate (*p* = 0.442), and PD nail bed (*p* = 0.124)

## Discussion

The nail apparatus is commonly affected in psoriasis, with varying prevalence rates reported in the literature [2], which can reach up to 90% over a person’s lifetime. However, nail manifestations are often overlooked in clinical practice, leading to aesthetic deformities, functional limitations, pain, decreased quality of life and overall well-being [10]. This burden of nail involvement emphasizes the importance of early diagnosis, given that nail psoriasis is believed to be a significant risk factor for developing psoriatic arthritis and serves as an important parameter for early diagnosis in these patients [11, 12]. 

In this study, the psoriasis Area and Severity Index (PASI) and Body Surface Area (BSA) were used as clinical metrics for evaluating patients. Patients in the psoriatic arthritis groups (groups 3 and 4) exhibited significantly lower PASI and BSA values compared to patients in the psoriasis groups without joint disease (groups 1 and 2). One possible explanation for this disparity is that patients with psoriatic arthritis often receive systemic treatment, resulting in better clinical control of cutaneous manifestations. The Nail psoriasis Severity Index (NAPSI) was used to assess the nail plate in groups with affected nails. However, no significant difference was observed between the groups without psoriatic arthritis (group 2) and those with psoriatic arthritis (group 4). Similar results were obtained in the evaluation of nails clippings in which it was not possible to determine whether the patient had arthritis or not based on the severity of microscopical findings [13]. 

In the present study ultrasound was employed as a method for characterizing the inflammatory process in patients with psoriasis and psoriatic arthritis.

Considering that the nail apparatus is linked to the musculoskeletal system and serves as a connection between the integument and the joint, ultrasound evaluation of nails emerged as a promising tool for early identification of nail psoriasis. It could potentially differentiate between patients with psoriasis and psoriatic arthritis patients. This study used parameters such as gray scale (GS) to visualize the nail plate’s trilaminar pattern and measure the nail bed, as well as Power Doppler (PD) to assess circulation and resistance index in the nail bed.

Patients with visible nail disease (groups 2 and 4) displayed a more pronounced loss of the trilaminar pattern compared to groups without nail disease (groups 1 and 3). Increased nail bed thickness is considered an early indicator of psoriatic nail disease, [14–15] a correlation supported by our study when correlating NAPSI with mean nail bed thickness (*p* = 0.0072) from the Spearman test (Table 1). However, when comparing groups with nail disease, including the patients with psoriasis and arthritis, no significant differences were noted concerning gray scale measurements. The degree of loss in the trilaminar pattern directly correlated with clinical nail involvement (NAPSI), though, being consistent with previous studies [10–12]. These findings align with De Rossi et al.‘s study [12], which also did not find a significant difference between patients with psoriasis, psoriatic arthritis, and the control group. A systematic review by Mendonça et al. [11] similarly found no statistically significant difference in nail bed thickening values between patients with psoriasis and psoriatic arthritis. The wide variation in measurements across the literature may be partly attributed to ethnic differences [12]. Our findings support that GS trilaminar disturbances seen in US is as good as clinical examination in the ability of discerning the presence or absence of dystrophy, but not in the diagnosing of inflammation in the enthesis.

Power Doppler assessment of the nail apparatus visualizes vascularization in the nail bed, revealing elongated, dilated, and tortuous vessels resulting from local inflammation. Increased blood flow in the nail bed directly correlates with disease activity. [16–17] While patients with psoriatic arthritis (groups 3 and 4) displayed similar vascular alterations in the nail bed, there were no significant differences observed in Power Doppler for patients with nail dystrophy (in the presence or absence of arthritis). The literature contains conflicting data on the utility of Doppler techniques in evaluating nail involvement in psoriasis and psoriatic arthritis [11]. This study’s results further emphasize these discrepancies. Mendonça et al.‘s [11] systematic review showed variable Power Doppler signals in both psoriasis and psoriatic arthritis patients, along with an observed increase in vascularity in healthy controls. Naredo et al. [18]., also unable to use the Power Doppler parameter to discriminate between control and psoriatic groups, or between clinically affected and non-clinically affected nails [18, 19]. 

Regarding the Power Doppler Resistance Index, no significant difference was noted between evaluated groups, consistent with De Rossi et al.‘s findings [12]. Some studies [20] indicate higher Resistance Index in psoriasis patients compared to controls, potentially due to endothelial dysfunction and capillaroscopy findings. In contrast, other studies show lower Resistance Index in psoriatic arthritis patients, possibly linked to inflammation extending to the nail apparatus [11]. These variations in Resistance Index findings reinforce the need for further evaluation in future studies to better determine how to apply the measure in clinical practice, including possible differences between specific subgroups.

The study’s limitations include single-examiner ultrasound assessments and patients undergoing clinical treatment, which may impact sonographic findings.

The choice to have a single examiner conduct the ultrasound assessments may introduce bias into the results. Different examiners may have slightly different approaches and interpretations, which could influence the study’s conclusions. The fact that patients were undergoing clinical treatment may affect sonographic findings. Depending on the type of treatment, whether medicinal or physiotherapeutic, ultrasound characteristics may be altered, and the results may not be generalizable to a broader population that includes those not undergoing treatment.

Further research is necessary to elucidate these nuances and refine the diagnostic utility of ultrasound in distinguishing between these conditions.

In conclusion, ultrasound, a cost-effective method, holds consistency between grayscale and power doppler abnormalities and nail dystrophy severity but fails to be useful in the differentiation between patients with or without arthritis.

## Electronic supplementary material

Below is the link to the electronic supplementary material.

Supplementary Material 1

Supplementary Material 2

## Data Availability

The datasets used and/or analyzed during the current study are available from the corresponding author on reasonable request.
