# Supplementary material for: Ultrasonographic and power doppler parameters of nails fail to differentiate between onychodystrophy in patients with psoriasis vulgaris or psoriatic arthritis
Source: Adv Rheumatol. 2024 Apr 11;64:25. doi: 10.1186/s42358-024-00367-x (PMC13488622; doi:10.1186/s42358-024-00367-x)
Supplement: Supplementary file 1 [file 42358_2024_367_MOESM2_ESM.pdf]

Dear Marcos Renato de Assis

Editor-in-Chief,

We are grateful to you for your time and to the Reviewer for the valuable comments and recommendations that will contribute to the quality of our study. We are pleased that you are considering our manuscript for publication. Below you will find the changes we have made in response to the Reviewer's recommendations. All revisions and insertions have been highlighted in yellow in the body of the text.

Reviewer #1:

Reviewer's suggestion:
1. Lines 40-41: "Subclinical inflammation detectable by US has been noted even in psoriasis patients lacking prior history of arthropathy or psoriatic arthritis" - please include a reference for this statement.

Response in agreement with the Reviewer
Dear Reviewer, this observation it is importance to our manuscript, and we thank you for your contribution. We apologize for forgetting and now, we have added a reference in the manuscript.

Reviewer's suggestion:
2. Line 52: "A cross-sectional study was conducted" - when? What is the timeframe? Please update the manuscript.

Response in agreement with the Reviewer:
Dear Reviewer, we agree with and appreciate your observation. A cross-sectional study was conducted from November 2020 to January 2022, this information about the research time is now inserted in the manuscript.

Reviewer's suggestion:
3. Line 54: "the outpatient Dermatology" - did you screen/include all the patients who came to this department within the timeframe, or just some of them? This is important for randomness; therefore, it should be explained in the manuscript.

Response in agreement with the Reviewer:
Dear Reviewer, thank you for your comment. Patients who were seen at the outpatient clinic, all were evaluated consecutively. Only those who met the inclusion criteria and agreed to participate in the research were included. Now it is explained in the manuscript.

.

Reviewer's suggestion:
4. Line 55: "Exclusion criteria were…" - did you enroll children or only adults? Please add in the manuscript the age cutoff for inclusion.

Response in agreement with the Reviewer:

Dear Reviewer, thank you for your comment. We did not include children, only adults aged 18 and above with no cut-off age. This information has been included in the manuscript.

Reviewer's suggestion:
5. Line 57: "corticosteroid" - the preferred term used in the literature is "glucocorticoid". Please update the manuscript.

Response in agreement with the Reviewer:
Dear Reviewer, we appreciate and have followed your suggestion.

Reviewer's suggestion:
6. Line 58: "diagnosis of osteoarthritis in the hands" - it is rare for people above 50 years of age not to have hand osteoarthritis, especially if they also have psoriatic arthritis. Have you really used this exclusion criterion? Please act accordingly.

Response in agreement with the Reviewer:
I agree with your technical explanation, but patients with a confirmed and documented diagnosis of osteoarthritis in medical records were really excluded.

Reviewer's suggestion:
7. Line 62: "Patients diagnosed with psoriasis and psoriatic arthritis were sequentially assessed" - did each patient have all his study procedures done on the same day? Please update the manuscript.

Response in agreement with the Reviewer:
Dear Reviewer, thank you for your comment.
All study procedures were not conducted on the same day. Clinical examination and collection of clinical information were performed on the same day. The ultrasound examination was scheduled on a different day according to the availability. This information has been included in the manuscript.

Reviewer's suggestion:
8. Line 63: "gender" - did you ask your patients what they consider themselves to be, or did you simply note the biological/genetic differentiation? In the latter case, "sex" is a more appropriate term for scientific and medical text.

Response in agreement with the Reviewer:
Dear Reviewer, thank you for your comment.
We simply notice the biological/genetic differentiation. In this case we changed the term in the manuscript as recommended

Reviewer's suggestion:
9. Line 66: "The study cohort comprised 120 participants, divided equally into four groups" - this is a piece of information from the Methods section, but it belongs to the Results section. Nobody knows how many patients will come to a clinic within a specific timeframe, unless they plan to stop at a certain number. So, either move the number of participants to the Results, or explain why you planned as a method to have 120 cases.

Response in agreement with the Reviewer:
Dear Reviewer, we appreciate and have followed your suggestion and move the number of participants to the results.

Reviewer's suggestion:
10. Line 125: please include in the statistics section how you reported your continuous variables. Since you used nonparametric tests, your countinuous variables were probably distributed non-normally, so you should state that you reported them as "median (minimum-maximum)" or "median (interquartile range)".

Response in agreement with the Reviewer:
Dear Reviewer, thank you for your comment.
The data were tested for their distribution using the Shapiro-Wilk test. Descriptive statistics - median (minimum-maximum) were employed to summarize the results involving quantitative variables, while categorical variables were expressed in terms of absolute and relative frequencies. We inserted this information into the manuscript.

Reviewer's suggestion:
11. Line 130: what software was used to do the statistical tests. Please cite it.

Response in agreement with the Reviewer:
It was used, Software Statstica Statsoft 7.0. we inserted this information into the manuscript.

Reviewer's suggestion:
12. Line 136: "Peripheral Arthritis, while 25% presented Axial Arthritis" - these nouns are common, they do not require an uppercase initial letter.

Response in agreement with the Reviewer:
Dear Reviewer, we appreciate and have followed your suggestion.

Reviewer's suggestion:
13. Line 142: "the Spearman test" - this test was not mentioned in the Statistics section. Please mention this test in the Statistics section.

Response in agreement with the Reviewer:
Now, it is mentioned in the statistical analysis section.

Reviewer's suggestion:
14. Table 1 - a table should be able to stand alone form the text, therefore please include in this table, any way you see fit, p value information.

Response in agreement with the Reviewer:
Dear Reviewer, we appreciate and have followed your suggestion.

Reviewer's suggestion:
15. Line 223: "The study's limitations…" - please end the article with its conclusions, not with limitation. Please relocate the limitation before the conclusion.

Response in agreement with the Reviewer:
Dear Reviewer, we appreciate and have followed your suggestion:
We moved the discussion of limitations from the text to before the conclusion.

Reviewer #2:

Reviewer's suggestion:
1-"A positive relationship was found with the average ultrasound thickness of the nail bed and the Nail Psoriasis Severity Index (NAPSI)by the spermann test (p = 0.0072)." No need to provide as "Spearman's" in here. Please use "correlation analysis" instead. Please give the rho value instead of p value.

Response in agreement with the Reviewer:
Dear Reviewer, we appreciate and have followed your suggestion. The rho value 0,344 is now cited in the manuscript and correlation analysis was used.

Reviewer's suggestion:
2-"(p = 0.44), nail bed PD (p =0.12)." 3 decimals are required.

Response in agreement with the Reviewer:
We appreciate your suggestion. Now, the third decimal place is included and cited in the manuscript.

p= 0,442
p= 0,124

Reviewer's suggestion:
3-A more detailed introduction is necessary.

Response in agreement with the Reviewer:
Dear Reviewer, we appreciate and have followed your suggestion:
In the introduction, we conducted a scientific exploration, delving beyond ultrasonography to discuss psoriasis, psoriatic arthritis, and their relationship with nail dystrophy.

Reviewer's suggestion:
4-The inclusion and exclusion criteria should be given more clearly.

Response in agreement with the Reviewer:

Dear Reviewer, thank you for your comment. The inclusion criteria were not clearly outlined in the manuscript. We have now clarified both the inclusion and exclusion criteria for the study.

Reviewer's suggestion:
5-ethical approval date and number should be given.

Response in agreement with the Reviewer:
Dear Reviewer, thank you for your comment. Now, the requested information is appropriately detailed in the manuscript in the corresponding section.
The research project was submitted to the Ethics Committee of the Mackenzie Evangelical Faculty of Paraná (CAAE: 36864720.4.0000.0103) and received a favorable opinion for the study to proceed under protocol 4.279.067 on September 15, 2020.

Reviewer's suggestion:
6-All devices used should be given along with country of origin and manufacturer.

Response in agreement with the Reviewer:
The device is the Mylabel40 model from the Esaote brand (Italy). We put the information in the manuscript.

Reviewer's suggestion:
7-A separate statistical analysis section is required.

Response in agreement with the Reviewer:
Dear Reviewer, we appreciate and have followed your suggestion:
All statistics have been double-checked.

Reviewer's suggestion:
8-Please provide exact names of statistical tests.

Response in agreement with the Reviewer:
Dear Reviewer, thank you for your comment.
The data were tested for their distribution using the Shapiro-Wilk test. Descriptive statistics - median (minimum-maximum) were employed to summarize the results involving quantitative variables, while categorical variables were expressed in terms of absolute and relative frequencies. For group comparisons, the Kruskal-Wallis test was employed for quantitative variables and Pearson Chi-square test for categoric variables. Correlation analysis was performed using the Spearman coefficient. The analyzes were carried out using the software Statstica Statsoft 7.0., considering a p-value < 0.05 to indicate statistical significance**.**
Reviewer's suggestion:
9-Which chi-squared test was used?

Response in agreement with the Reviewer:
The data were tested for their distribution using the Shapiro-Wilk test. Descriptive statistics - median (minimum-maximum) were employed to summarize the results involving quantitative variables, while categorical variables were expressed in terms of

absolute and relative frequencies. For group comparisons, the Kruskal-Wallis test was employed for quantitative variables and **Pearson Chi-square test for categoric variables**. Correlation analysis was performed using the Spearman coefficient. The analyzes were carried out using the software Statstica Statsoft 7.0., considering a p-value < 0.05 to indicate statistical significance

Reviewer's suggestion:
10-Decimals should not be presented with comma.

Response in agreement with the Reviewer:
Dear Reviewer, we appreciate and have followed your suggestion:
We corrected and removed the commas that were present in the table.

Reviewer's suggestion:
11-The limitations of the study should be discussed.

Response in agreement with the Reviewer:
Dear Reviewer, we appreciate and have followed your suggestion:
We deepened the discussion on the study's limitations, and it has been included in the manuscript.

Reviewer's suggestion:
12-Reference list should be diversified.

Response in agreement with the Reviewer:
Dear Reviewer, we appreciate and have followed your suggestion.

Reviewer's suggestion:
13-table presentations should be added.

Response in agreement with the Reviewer:
Dear Reviewer, we appreciate and have followed your suggestion. We have included tables in the manuscript.

Again, we would like to thank you for considering our manuscript for publication in the Advances in Rheumatology

Cordially,
Anber Tanaka
