# Supplementary material for: Ultrasonographic and power doppler parameters of nails fail to differentiate between onychodystrophy in patients with psoriasis vulgaris or psoriatic arthritis
Source: Adv Rheumatol. 2024 Apr 11;64:25. doi: 10.1186/s42358-024-00367-x (PMC13488622; doi:10.1186/s42358-024-00367-x)
Supplement: Supplementary file 2 [file 42358_2024_367_MOESM1_ESM.docx]

**List of abbreviations**

US: Ultrasound; DIP: distal interphalangeal; PASI: Psoriasis Area and Severity Index; NAPSI: Nail psoriasis Severity Index; GD: gray-scale; PD: Power Doppler; PRF: Pulse Repetition Frequency; BSA: Body Surface Area.
